# Genome-wide Association Study of Susceptibility to Respiratory Syncytial Virus Hospitalization in Young Children <5 Years of age

**DOI:** 10.1093/infdis/jiad370

**Published:** 2023-09-04

**Authors:** Amanda Marie Egeskov-Cavling, Maarten van Wijhe, Victor Yakimov, Caroline Klint Johannesen, Andrew J Pollard, Ramona Trebbien, Jonas Bybjerg-Grauholm, Thea Kølsen Fischer, for the Respiratory Syncytial Virus Consortium in Europe (RESCEU) investigators, Harish Nair, Harry Campbell, Philippe Beutels, Louis Bont, Andrew Pollard, Peter Openshaw, Federico Martinon-Torres, Terho Heikkinen, Adam Meijer, Thea Fischer, Maarten van den Berge, Carlo Giaquinto, Michael Abram, Kena Swanson, Bishoy Rizkalla, Charlotte Vernhes, Scott Gallichan, Jeroen Aerssens, Veena Kumar, Eva Molero

**Affiliations:** Department of Virus and Microbiological Special Diagnostics, Statens Serum Institut, Copenhagen; Department of Clinical Research, Nordsjællands Hospital, Hilleroed; Department of Virus and Microbiological Special Diagnostics, Statens Serum Institut, Copenhagen; Department of Science and Environment, Roskilde University; Neonatal Genetics, Statens Serum Institut, Copenhagen, Denmark; Department of Virus and Microbiological Special Diagnostics, Statens Serum Institut, Copenhagen; Department of Clinical Research, Nordsjællands Hospital, Hilleroed; Oxford Vaccine Group, Department of Pediatrics, University of Oxford and National Institute for Health and Care Research Oxford Biomedical Research Centre, United Kingdom; Department of Virus and Microbiological Special Diagnostics, Statens Serum Institut, Copenhagen; Neonatal Genetics, Statens Serum Institut, Copenhagen, Denmark; Department of Virus and Microbiological Special Diagnostics, Statens Serum Institut, Copenhagen; Department of Clinical Research, Nordsjællands Hospital, Hilleroed; Department of Public Health, University of Copenhagen, Denmark

**Keywords:** RSV, genetic association studies, genome-wide association study, respiratory syncytial virus

## Abstract

**Background:**

Worldwide, respiratory syncytial virus (RSV) infections are among the most common causes of infant hospitalization. Host genetic factors influencing the risk and severity of RSV infection are not well known.

**Methods:**

We conducted a genome-wide association study (GWAS) to investigate single-nucleotide polymorphisms (SNPs) associated with severe RSV infections using a nested case-control design based on 2 Danish cohorts. We compared SNPs from 1786 children hospitalized with RSV to 45 060 controls without an RSV-coded hospitalization. We performed gene-based testing, tissue enrichment, gene-set enrichment, and a meta-analysis of the 2 cohorts. Finally, an analysis of potential associations between the severity of RSV infection and genetic markers was performed.

**Results:**

We did not detect any significant genome-wide associations between SNPs and RSV infection or the severity of RSV. We did find potential loci associated with RSV infections on chromosome 5 in 1 cohort but failed to replicate any signals in both cohorts.

**Conclusions:**

Despite being the largest GWAS of severe RSV infection, we did not detect any genome-wide significant loci. This may be an indication of a lack of power or an absence of signal. Future studies might include mild illness and need to be larger to detect any significant associations.

## Teaser

We conducted a genome-wide association study (GWAS) to investigate the association between the severity of RSV infection and genetic markers. Despite this being the largest GWAS of severe RSV infection, we did not detect any genome-wide significant loci.

Worldwide, respiratory syncytial virus (RSV) infections are among the most common causes of infant hospitalization and are responsible for a significant burden of infant mortality [1, 2]. Several risk factors for RSV infection are already known, namely, premature birth, lung and heart diseases, and immunodeficiency [3, 4]. These risk factors do not fully explain the variation in disease susceptibility or severity of the disease because the majority of the infants diagnosed with RSV are otherwise healthy, with no clinical risk factors for severe disease [1, 5]. Therefore, host genetic factors might help to explain disease etiology of RSV infections as well as the severity of the disease [5, 6].

The genetic susceptibility of RSV infections has been found to be a complex trait with many different host genetic variants influencing the risk for RSV infections [5–7]. Several studies have suggested a genetic association between severe RSV infections and single-nucleotide polymorphisms (SNPs) in genes associated with innate host defense, cytokine or chemokine response, and altered Th1/Th2 immune responses [6, 8]. A genome-wide association study (GWAS) as an approach has been used widely to associate specific genetic variations with specific diseases [9]. The approach includes scanning thousands of SNPs from different individuals to find genetic markers that can be used to predict the manifestation of a disease.

In recent years, GWAS has successfully identified host–pathogen associations between SNPs and diseases such as asthma, chronic obstructive pulmonary disease (COPD), coronavirus disease 2019 (COVID-19), tuberculosis, and meningococcal diseases [9–11]. Compared with asthma, COPD, and COVID-19, very few genetic studies have examined the severity or susceptibility to RSV infections using GWAS. A GWAS study by Pasanen et al that was based on a Finnish-Swedish population of 217 children hospitalized for bronchiolitis identified several suggestive association signals in SNPs (rs269094, rs9591920, rs1537091), but they did not find any significant genome-wide association [5]. The rate and scale of discovery in the genetic studies of RSV infections have been limited by sample sizes [5, 8, 12, 13]. A more comprehensive and larger GWAS of RSV infections could help to identify the genetic contributions to the susceptibility and severity of RSV infections and recognize risk groups to ultimately propose targets for the primary prevention of RSV infection and new approaches for vaccine strategies. This study aims to alleviate this shortcoming by investigating SNPs associated with severe RSV disease within 2 large case-cohort studies including >60 000 biobank samples.

## METHODS

### Source Population

We performed a GWAS using a nested case-control design based on 2 comprehensive cohorts, namely iPSYCH2012 [14] and iPSYCH2015 [15]. In total, these cohorts comprise 134 230 individuals. The cases of these cohorts were selected as patients with a registered psychiatric diagnosis of interest (for further description of the diagnoses, see Pedersen et al and Bybjerg-Grauholm et al [14, 15]). The controls were randomly selected among the entire Danish population with the only inclusion criteria being born to a mother with a Danish social security number and being alive and residing in Denmark on their first birthday. Psychiatric status was not considered in selecting controls, ensuring an unbiased control population. Individuals from iPSYCH2012 and iPSYCH2015 were linked to Danish registry data by using a unique personal identifier, to extract information on hospitalizations and demographic information.

We extracted information from the Danish National Patient Register (DNPR), Medical Birth Register (MBR), and the Civilian Registration System (CRS). DNPR is a longitudinal register covering all of Denmark and contains data on all hospital admissions since 1977, including *International Classification of Diseases, Tenth Revision* (*ICD-10*) discharge codes since 1995. Birth and maternal data are contained within the MBR. The CRS includes demographic and residence information for all inhabitants. Across all 3 registers, we had information from 1995 through 10 October 2018.

### Study Population

**Figure 1.**
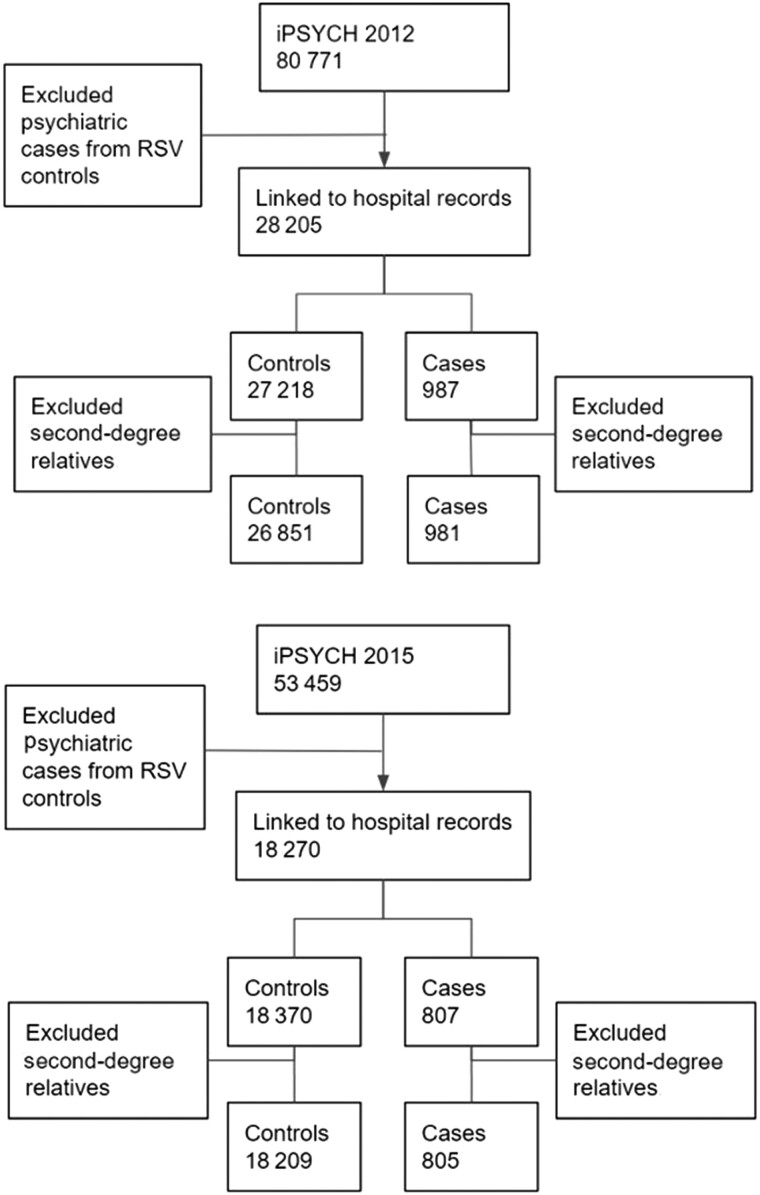
Flow diagram for the study design and population—iPSYCH2012 and iPSYCH2015. Abbreviation: RSV, respiratory syncytial virus.

RSV cases were obtained from both iPSYCH populations and their respective controls and cases. The hospitalized RSV cases were identified from DNPR, using *ICD-10* codes. We applied the following *ICD-10* code to identify both primary and secondary diagnoses: J12.1, J20.5, J21.0, and B97.4. Before *ICD-10*, no RSV-specific *ICD* codes existed. As such, only patients with a hospital admission coded with an RSV-associated *ICD-10* code after 1995 were included as cases. To retain as many RSV cases as possible, we did not restrict the cohort to children born within the *ICD-10* period and included all children aged <5 years with an RSV-related hospital admission. As all the included cases have been hospitalized with an RSV diagnosis, cases likely had a severe RSV infection, although we lacked clinical information on severity. In an additional analysis, severe RSV cases were defined by proxy as a length of hospital stay of >2 days. The controls were obtained from only the iPSYCH controls, which were randomly selected among the entire Danish population and did not have an RSV-related hospitalization recorded. To limit the loss of cases, cases and controls were not matched. See Figure 1 for the study design and selection of the population.

### DNA Extraction

The DNA was extracted and amplified from the neonatal dried blood spot samples obtained from the Danish Neonatal Screening Biobank. The methods used for DNA extraction in iPSYCH2012 and iPSYCH2015 are described in detail within the respective papers and briefly recapped here [14, 15]. A different experimental setup was used for the 2 cohorts. All steps from the laboratory through initial association were done independently and results were not merged until the final meta-analysis.

DNA extraction and whole genome amplification were done according to the method described by Hollegaard et al [16]. The amplified DNA was then genotyped with an Illumina Array (Illumina, California, San Diego). In iPSYCH2012 the PsychChip was used, while iPSYCH2015 used the Global Screening Array version 2. Variants in iPSYCH2012 were called using a merge of 3 different call trainings; GenCall, Birdseed, and zCall according to the order described in the iPSYCH2012 paper. iPSYCH2015 variants were trained exclusively using Gentrain version 3. The cohorts were imputed using Ricopoli [17]; the processing is described in detail elsewhere [18]. In short, a thorough pre-Impute QC was done to ensure data consistency after which prephasing/imputation was done using EAGLE version 2.3.5 [19] and Minimac [20]; the imputation reference used is Haplotype Reference Consortium version 1.0 [19].

### Statistical Analysis

Prior to performing genome-wide association testing, we filtered on minor allele frequency of 0.05, genotype missingness of 0.01, and Hardy–Weinberg equilibrium of 1e-6. We calculated the pairwise relatedness of all samples and excluded samples—preferentially controls—with King coefficient of 0.125, which corresponds to second-degree relatives using PLINK2 version 2.3. We calculated the first 10 principal components on linkage disequilibrium–pruned genotypes, which we used covariates in genome-wide association tests done on best-guess genotypes as a logistic regression assuming an additive genetic relation in PLINK2 version 2.3 [21]. The 2 iPSYCH cohorts were merged into a single meta-analysis using METAL [22] (version 2020-05-05). We performed gene-based testing, tissue enrichment, and gene-set enrichment using FUMA [23]. We compared our results to previously published results by either comparing the locus directly, in case we had it in our imputation panel, or by comparing it to the locus that was physically closest [5, 8, 24, 25].

In a separate analysis, we focused on potential associations with the severity of RSV disease among the hospitalized cases, hypothesizing that a stronger genetic signal might be present among more severely ill patients representing an underlying predisposition through immune system–associated genetic markers or airway-related genetics. As all the included cases were hospitalized RSV cases, all cases were to some extent severe. Since our data are based on hospital admission data, we used the length of hospital stay as our only parameter for disease severity. A hospital stay of >2 days was defined as the threshold for severe disease, and moderate cases were defined as a length of hospital stay of <2 days. This threshold for the severe cases was applied based on the argumentation that staying overnight at the hospital and the duration of the stay are closely related to the severity of the disease. However, the 2-day threshold was applied, instead of a threshold of <1 day, because only 10% of the RSV cases in both cohorts were admitted for only 1 night. The lengths of stay for the 2 cohorts are shown in Supplementary Figures 1 and 2.

Last, to investigate concordance with genetic loci previously suspected to be associated with RSV, we compared our results to loci reported as significant or “approaching significance” in the literature [5, 8, 24, 25].

## RESULTS

The iPSYCH cohorts consist of 66% and 60% cases (patients with a registered psychiatric diagnosis of interest); to maximize the number of RSV cases, the cases were drawn from both iPSYCH cases and controls (the iPSYCH cohorts and the diagnosis are further described in Pedersen et al and Bybjerg-Grauholm et al [14, 15]). Respectively, 83% and 82% of RSV cases from iPSYCH2012 and iPSYCH2015 were drawn from the iPSYCH cases. A total of 981 RSV cases and 26 851 controls were included from the iPSYCH2012 cohort, and 805 RSV cases and 18 209 controls were included from iPSYCH2015.

**Figure 2.**
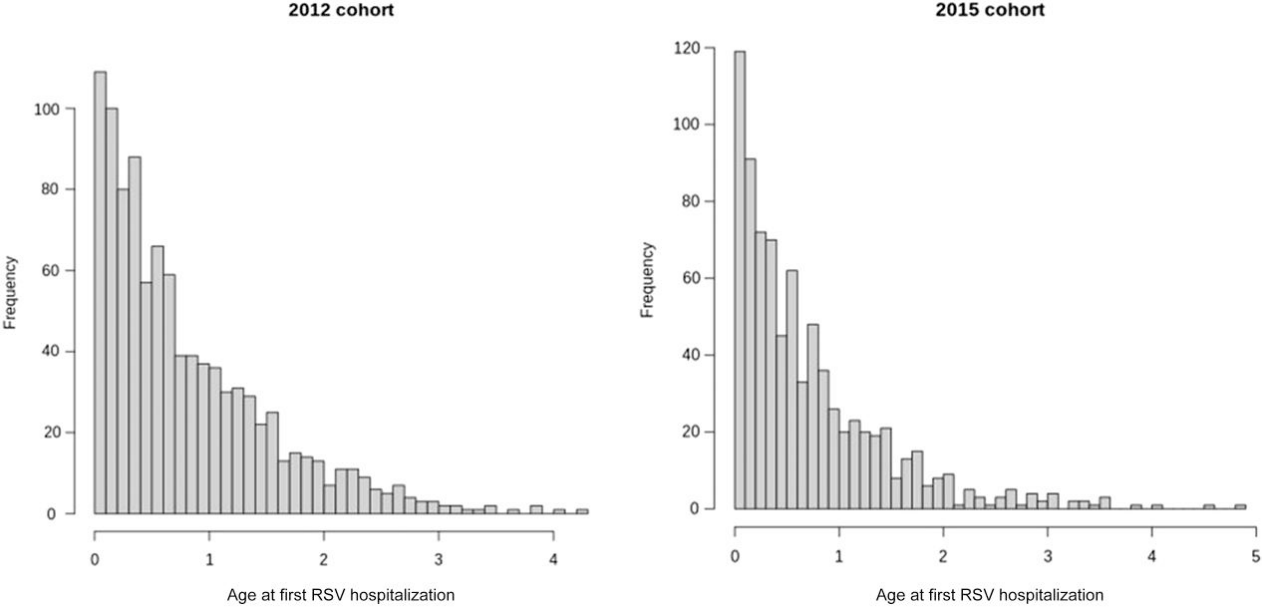
Age at first respiratory syncytial virus hospitalization—iPSYCH2012 and iPSYCH2015.

The median age at first RSV hospitalization was 230 days (interquartile range [IQR], 108–460 days) for the iPSYCH2012 cohort and 202 days (IQR, 87–390 days) for the iPSYCH2015 cohort. The characteristics of the 2 cohorts were very similar (see the distribution of age at first RSV hospitalization for the 2 cohorts in Figure 2). For both cohorts, the proportion of males was higher among the cases (67.8% and 60.0%) compared to the control groups (51.7% and 50.5%). As expected, prematurity, low birth weight, and maternal smoking were observed more frequently among RSV cases. The baseline characteristics are summarized in Table 1. For the additional analysis, we identified 769 severe cases hospitalized for >2 days and 212 moderate cases hospitalized for <2 days in iPSYCH2012; for iPSYCH2015, 622 severe cases and 183 moderate cases were identified.

**Table 1.** Descriptive Characteristics of Cases and Controls in the iPSYCH2012 and iPSYCH2015 Cohorts

Characteristic	iPSYCH2012		iPSYCH2015	
	RSV Cases	Controls	RSV Cases	Controls
Sex				
Male	666 (67.9)	13 283 (51.7)	483 (60)	8430 (50.5)
Female	315 (32.1)	12 425 (48.3)	322 (40)	8241 (49.5)
Birth length, cm, median (IQR)	52 (50–53)	52 (50–53)	52 (50–53)	52 (50–54)
Birth weight, g, median (IQR)	3430 (3010–3820)	3500 (3170–3850)	3400 (2900–3760)	3510 (3200–3860)
Prematurity				
Gestational age <37 wk	119 (12.4)	1111 (4.4)	112 (14.2)	746 (4.5)
Missing	25	1541	17	1806
Maternal smoking				
Nonsmoker	452 (55.3)	11 038 (75.1)	387 (55.7)	8161 (77.6)
Smoker	355 (43.5)	3463 (23.6)	294 (42.3)	2200 (20.9)
Stopped during pregnancy	10 (1.2)	191 (1.3)	14 (2.0)	159 (1.5)
Missing	164	12 181	110	7688
Maternal age, y, median (IQR)	28 (25–31)	28 (25–32)	28 (25–32)	29 (25–32)
Ethnicity (origin of parents)				
Danish	924 (93.8)	24 020 (93)	772 (95.7)	15 517 (92.6)
Non-Danish	57 (6.2)	1688 (7.0)	33 (4.3)	1154 (7.4)
Age at first RSV hospitalization, d, median (IQR)	230 (107–453)	…	202 (86–384)	…
Length of hospital stay, d, median (IQR)	4 (2–7)	…	3 (2–7)	…

Data are presented as No. (%) unless otherwise indicated.

Abbreviations: IQR, interquartile range; RSV, respiratory syncytial virus.

After correction for multiple testing, we failed to find any significant genetic associations in either the iPSYCH2012 or iPSYCH2015 cohorts (Supplementary Figures 3 and 4). In the iPSYCH2012 cohort, what appears to be a signal in chromosome 5 was detected—some of the related SNPs (rs79069767, rs11954156, and rs34341914) were located near gene TRIM36. However, this association failed to replicate in the iPSYCH2015 cohort. Quantile-quantile plots of the *P* values from the analysis can be seen in Supplementary Figures 5 and 6.

**Figure 3.**
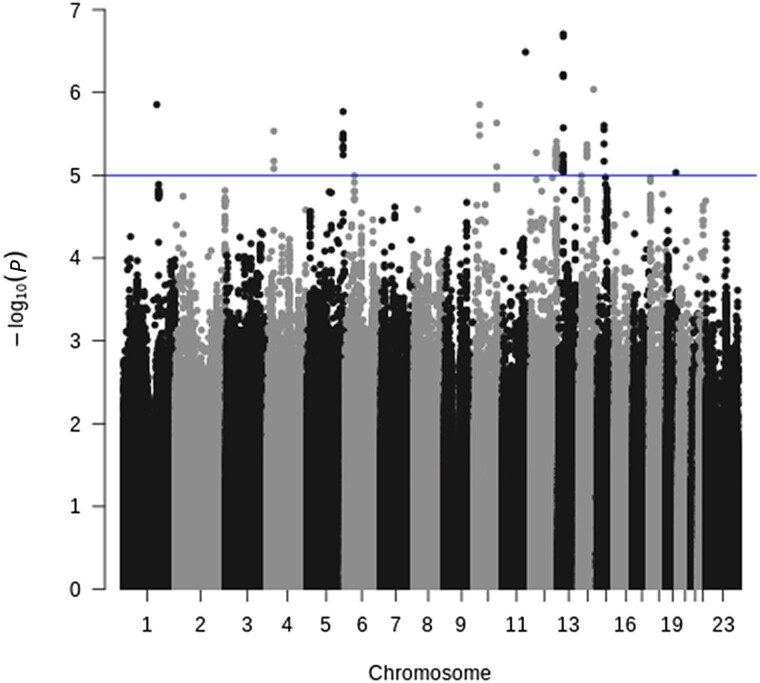
Manhattan plot of meta-analyses of the iPSYCH2012 and iPSYCH2015 genome-wide association study analysis.

To see more details on possible replicates in the 2 cohorts, we compared *P* values as well as odds ratios between them, as seen in Supplementary Figures 7 and 8. We did not observe any correlation between odds ratios or *P* values in the 2 cohorts, which is consistent with a lack of detectable signal. We also performed a meta-analysis of the 2 GWAS analyses (Figure 3), which also did not show any consistent signal. Upon performing gene-set enrichment analysis (Supplementary Tables 1 and 2), there was no correspondence between enriched gene sets of the 2 cohorts or any gene sets with an obvious biological connection to respiratory disease (such as lung or immune-related systems). Tissue expression analysis similarly did not replicate between the 2 cohorts, nor do any tissues with an obvious biological connection to respiratory disease appear significant (Supplementary Figures 9 and 10)—again, concordant with a lack of signals in the original association analysis. Performing gene-wise association testing (Supplementary Figures 11 and 12) did not increase signal in either the iPSYCH2012 or the iPSYCH2015 cohort.

In the separate analysis, we stratified the phenotype into severe and moderate disease, to analyze the severity based on the length of hospital stay (eg, a severe case was defined as a hospitalization length of >2 days and a moderate case was defined as a hospitalization length of <2 days). In the analysis comparing the severe cases, moderate cases, and controls, no significant associations between SNPs and RSV disease severity were found in any of the 2 cohorts or the meta-analysis of both (Supplementary Figures 13–15). For future studies, the top 10 associated SNPs among severe cases for both cohorts are presented in Supplementary Tables 3 and 4.

Last, compared to genetic loci previously associated with RSV, we found no concordance (Table 2). There are no SNPs in common between the top 10 associated SNPs among severe cases in either cohort (Supplementary Tables 3 and 4) and the suggestive SNPs found in other literature (Table 2).

**Table 2.** *P* Values in the iPSYCH2012 and iPSYCH2015 Genome-wide Association Study Analysis of Loci Found Significant in Literature

rsid_list	rsid_obs	*P* Value (2012)	*P* Value (2015)	Gene Name	Ensembl Gene Name
rs643070	rs643070	.392721	.432524	…	…
rs10757212	rs10757212	.793188	.205008	…	ENSG00000147873
rs62452699	rs62452699	.730056	.268024	…	…
rs14701	rs62519393	.819967	.66778	SLC22A5	ENSG00000197375
rs274549	rs274549	.32004	.335202	SLC22A5	ENSG00000197375
rs734244	rs734244	.825872	.501305	…	ENSG00000113520
rs2057768	rs2057768	.257738	.229813	…	…
rs2243250	rs2243250	.891141	.63742	…	…
rs2243270	rs2243270	.873168	.966624	…	ENSG00000113520
rs1805011	rs1805011	.47974	.0230317	…	ENSG00000077238
rs2070874	rs2070874	.969218	.679197	…	ENSG00000113520
rs2137526	rs2137526	.303116	.0107312	…	…
rs3794628	rs3794628	.627582	.0183072	…	ENSG00000260630
rs4976604	rs11740649	.0597445	.334828	…	ENSG00000113645
rs7727544	rs7727544	.197231	.110784	SLC22A4	ENSG00000072682
rs231216	rs231216	.631026	.886473	…	ENSG00000167595
rs76079505	rs76079505	.229017	.899119	…	…
rs3093457	rs3093457	.462514	.788559	…	ENSG00000124334
rs798112	rs61141728	.603815	.526968	…	…
rs1060826	rs1060826	.977795	.335117	…	ENSG00000007171
rs113409681	rs113409681	.982263	.527881	…	…
rs1001338	rs11535866	.568534	.768906	…	…
rs17009617	rs17009617	.910381	.772564	C10orf71	…
rs76728164	rs76728164	.581287	.346692	…	…
rs111255454	rs111255454	.619039	.873864	…	ENSG00000150760
rs2407992	rs2407992	.815097	.627092	…	ENSG00000101916
rs1914408	rs1914408	.936438	.616536	…	ENSG00000115415
rs1914408	rs1914408	.936438	.616536	…	LRG_111
rs3116494	rs3116494	.841091	.416455	ICOS, CD28, CTLA4	ENSG00000178562
rs11886348	rs7588045	.497315	.975321	…	…
rs9591920	rs9591920	.891924	.758182	…	…
rs142670120	rs1453905	.933224	.697814	…	…
rs235326	rs235326	.97303	.518998	ITGB2	ENSG00000160255
rs235326	rs235326	.97303	.518998	ITGB2	LRG_76
rs117674297	rs117674297	.290762	.203401	…	…
rs2254514	rs2254514	.770518	.806528	…	ENSG00000164136
rs4697072	rs4697072	.626257	.651547	…	…
rs935	rs2814858	.59923	.100688	…	ENSG00000223865
rs1799724	rs1799724	.367301	.433579	TNF	…
rs1047985	rs9272426	.444814	.228809	…	ENSG00000196735
rs7747909	rs7747909	.985351	.0526485	…	ENSG00000112115
rs72837837	rs35302816	.712254	.384372	…	ENSG00000008083
rs379083	rs379083	.416746	.0557451	…	…
rs574174	rs574174	.65046	.350884	…	ENSG00000149451
rs4010971	rs4805334	.863029	.745247	…	…
rs1441586	rs1441586	.68887	.121685	…	ENSG00000149534
rs149234067	rs4713676	.626185	.241942	…	ENSG00000205177
rs149234067	rs4713676	.626185	.241942	…	ENSG00000085063
rs6437736	rs6437736	.203739	.795273	…	…
rs529417345	rs34506014	.924478	.495147	…	ENSG00000145113
rs201623571	rs34506014	.924478	.495147	…	ENSG00000145113
rs548345415	rs34506014	.924478	.495147	…	ENSG00000145113
rs36086140	rs191651	.202755	.949223	…	ENSG00000114790
rs56039226	rs4723837	.0433807	.518616	…	ENSG00000168329
rs11688	rs11688	.673798	.976841	…	ENSG00000177606
rs1041163	rs1041163	.795596	.162681	…	…
rs1800872	rs1800872	.920832	.626165	IL10, IL19	…
rs2251746	rs2251746	.892346	.301794	FCER1A	ENSG00000179639
rs2274064	rs2274064	.725517	.753861	NCF2	ENSG00000116698
rs2274064	rs2274064	.725517	.753861	NCF2	ENSG00000116701
rs2274064	rs2274064	.725517	.753861	NCF2	LRG_88
rs61202512	rs61202512	.828077	.877728	…	…
rs79062720	rs77506558	.236349	.429427	…	…

## DISCUSSION

In this study, we performed a genome-wide association study to investigate genetic SNPs associated with severe RSV disease (eg, hospitalization length of >2 days). We applied a nested case-control design based on 2 comprehensive cohorts with a total of 1786 children hospitalized with a RSV diagnosis and 45 060 controls, namely iPSYCH2012 and iPSYCH2015. This currently represents the largest sample size for a GWAS investigating SNPs associated with RSV infections.

We did not detect any significant genome-wide associations between genetic SNPs and RSV infections but did identify suggestive loci associated with RSV infections on chromosome 5 (SNPs: rs79069767, rs11954156, rs34341914) located near gene TRIM36 in the iPSYCH2012 cohort. However, we were not able to replicate this suggestive association in the iPSYCH2015 cohort. Despite the lack of replication within both cohorts, these signals might reflect true associations, but further investigation of these loci did not provide biological plausible explanations. One explanation for the differences in the 2 cohorts can relate to the RSV strains, where 1 RSV strain may have been more dominant in 1 of the cohorts compared to the other. However, the analysis of the RSV strain pathogenicity interacting with the host genetics was not taken in this study. Based on our sample size, we expected to be able to detect any strong signals or associations between any genetic markers and severe RSV infection. Yet, our study might have been underpowered to find weaker associations between genetic markers and severe RSV disease, so any real effect or associations of genetics on RSV hospitalizations can be suggested to be weak if present.

In our GWAS we also explored the association between SNPs and the severity among our case population of hospitalized RSV cases. Severe RSV cases were defined as being hospitalized for >2 days compared to moderate RSV cases with a hospitalization length of <2 days. With the hypothesis that genetic effects may be stronger among those predisposed to more severe respiratory infections, we did not find any suggestive SNPs; therefore, nongenetic differences between the severe cases of RSV likely dominated any genetic associations. This study is limited by the definition of disease severity because all included RSV cases were hospitalized and can be argued to be severe. Another way for future studies to make a more accurate distinction between severe and moderate hospitalized RSV cases could be to compare the top quartile of the number of days of admission with the bottom quartile, albeit it will require a larger number of cases than this study. However, the most essential would be to investigate RSV disease severity and host genetic risk factors by comparing hospital cases with nonhospital cases to be able to explore any genetic effect in severe cases. Additionally, it could be recommended to measure RSV disease severity based on 1 or more other parameters such as intensive care unit admission, oxygen supplementation, or respiratory rate.

The main limitation of our study is the focus on children admitted to hospitals with RSV infection. We did not include RSV infections not requiring hospitalization, as these are not recorded in the DNPR or any other register in Denmark. While the focus of this study may have had the potential to look only at strong genetic risk factors, it has the downside of limiting the number of cases as well as biasing any potential findings. Cases admitted to the hospital due to RSV might have genetic risk factors that predispose them to any severe respiratory infections, not just RSV. Any findings might thus not be disease-specific. Furthermore, we did not adjust for other severe comorbidities such as metabolic or neurological diseases and syndromes. This may have lowered the power to detect any genetic effects but would also have lowered the number of cases included. Since we found no indication of any genetic markers replicating in both cohorts, it is unlikely that this limitation has any strong influence on our results.

We also compared our findings with signals and genetic loci found in other studies. A GWAS study based on a Finnish-Swedish population did not detect any significant GWAS association with RSV infections either. However, several suggestive association signals were detected near the region near LOC105375265 and LOC105375266 in chromosome 7 and the genes VSTM4, C10orf71, and DRGX in chromosome 10 [5]. Other genetic association studies have successfully identified SNPs associated with susceptibility and severe RSV infections and both linked RSV disease severity to innate immunity—respectively, the innate immune genes VDR (rs10735810; *P* = .0017) and the IL13–IL4 locus in the 5q31 cytokine cluster [8, 24]. Compared with the GWAS approach, the methods used in these studies are based on analyses of preselected SNPs and do not have the same untargeted approach. A strength of our study is that GWAS is an untargeted hypothesis-free approach. The approach is based on no preconceptions about the pathogenesis of disease and therefore has the potential to detect new findings. We were, however, not able to replicate these previous findings and we found noncorrelation to the SNPs detected in these studies.

The GWAS genotypes hundreds of thousands of SNPs for each individual. These analyses require corrections of numerous comparisons, and a large study population is needed to outweigh the background noise due to variations in the human population. Therefore, these types of studies have a stringent threshold for statistical significance, which also means that statistical power to detect effects is often lacking. That our study did not find genome-wide significant loci, nor replicated suggestive findings, is an indication of a lack of power or an absence of signal. Nevertheless, with our study being the largest and most comprehensive GWAS on RSV currently, we should expect to have had sufficient statistical power to discover genetic variants contributing to the severity of RSV infections. Furthermore, based on a randomly selected cohort from the entire Danish population, our results should be unbiased and able to estimate the effect size of genetic markers of severe RSV infections. Albeit the results of this GWAS are negative, the importance of the results should be highlighted based on the sample size and design of the study.

The iPSYCH cohorts consist of 66% and 60% cases (patients with a registered psychiatric diagnosis of interest); to maximize the number of RSV cases, the cases were drawn from both iPSYCH cases and controls. Respectively, 83% and 82% of RSV cases from iPSYCH2012 and iPSYCH2015 were drawn from the iPSYCH cases, which indicates a small overrepresentation of patients with psychiatric disorders among RSV cases. One of the explanations for the overrepresentation of iPSYCH cases among RSV cases may be due to the acknowledged association between exposure to early-life infections and psychiatric disorders later in life [26]. However, the association is mainly interesting from a psychiatry perspective, since it is the RSV infection in children influencing the risk of psychiatric disorders later in life and not the other direction. Still, our results should be unbiased estimates of population genetic risk factors for RSV and provide results that are representative of the Danish population.

The median age at hospitalization in our study is slightly higher than other studies have found [27–29]. A clear explanation for this is lacking, although part of it lies in the inclusion of cases born prior to the start of RSV *ICD* coding in 1995, which artificially inflates the age at first RSV diagnosis. Another explanation might be the overweighting of cases from the iPSYCH cases, who may be more prone to visit the hospital and thus be diagnosed, and thereby also receive diagnoses later in life. Regardless, the difference with other studies is not large, and given that no genetic signals were even remotely replicated in the second cohort, is unlikely to have had a significant impact on our results.

## CONCLUSIONS

In conclusion, our GWAS study did not find any significant genetic SNPs associated with RSV infections requiring hospitalization or the severity of the disease. The lack of any significant findings is an indication of a lack of power or absent or weak signals. Although this study is still the largest GWAS on RSV disease conducted, the result of the study and the large body of data should benefit future studies. Larger cohorts are needed to detect any significant associations, validate the current suggestive signals, and discover genetic variations in the susceptibility to RSV. Investigating RSV disease severity and host genetic risk factors by comparing hospital cases with nonhospital cases to be able to explore any genetic effect in severe cases would be essential for future research to investigate.

## Supplementary Data

Supplementary materials are available at *The Journal of Infectious Diseases* online. Consisting of data provided by the authors to benefit the reader, the posted materials are not copyedited and are the sole responsibility of the authors, so questions or comments should be addressed to the corresponding author.

## Supplementary Material

jiad370_Supplementary_Data

## Notes

***Acknowledgments***. The RESCEU investigators are as follows: Harish Nair (University of Edinburgh), Harry Campbell (University of Edinburgh), Philippe Beutels (Universiteit Antwerpen), Louis Bont (University Medical Center Utrecht), Andrew Pollard (University of Oxford), Peter Openshaw (Imperial College London), Federico Martinon-Torres (Servicio Galego de Saude), Terho Heikkinen (University of Turku and Turku University Hospital), Adam Meijer (National Institute for Public Health and the Environment), Thea Fischer (Statens Serum Institut), Maarten van den Berge (University of Groningen), Carlo Giaquinto (PENTA Foundation), Michael Abram (AstraZeneca), Kena Swanson (Pfizer), Bishoy Rizkalla (GlaxoSmithKline), Charlotte Vernhes (Sanofi Pasteur), Scott Gallichan (Sanofi Pasteur), Jeroen Aerssens (Janssen), Veena Kumar (Novavax), and Eva Molero (Team-It Research).

***Disclaimer.*** The funder had no role in the study design, data collection, data analysis, data interpretation, or writing of the report. This manuscript represents the views of the authors only. The European Commission is not responsible for any use that may be made of the information it contains.

***Financial support***. This project is part of the Respiratory Syncytial Virus Consortium in Europe (RESCEU). RESCEU was supported by the Innovative Medicines Initiative 2 Joint Undertaking (grant number 116019), which in turn receives support from the European Union's Horizon 2020 Research and Innovation Programme and the European Federation of Pharmaceutical Industries and Associations.
