# Supplementary material for: Genome-wide Association Study of Susceptibility to Respiratory Syncytial Virus Hospitalization in Young Children <5 Years of age
Source: J Infect Dis. 2023 Sep 4;230(2):e333–41. doi: 10.1093/infdis/jiad370 (PMC11326809; doi:10.1093/infdis/jiad370)
Supplement: jiad370_Supplementary_Data [file jiad370_supplementary_data.zip › Suppl_Figure Captions and Tables_Egeskov-Cavling et al.docx]

**Supplements**

**Figures captions:**

**Figure S1: Length of stay in hospital during RSV admission for the IPSYCH2012 cohort**

**Figure S2: Length of stay in hospital during RSV admission for the IPSYCH2015 cohort**

**Figure S3: Association testing of RSV in the iPSYCH2012 cohort.** The blue horizontal line represents the suggestive significance threshold of p-value = 1.0 × 10 −5.

**Figure S4: Association testing of RSV in the iPSYCH2015 cohort.** The blue horizontal line represents the suggestive significance threshold of p-value = 1.0 × 10 −5.

**Figure S5: Quantile-quantile plot of p-values in the iPSYCH2012 association analysis**

**Figure S6: Quantile-quantile plot of p-values from the iPSYCH2015 association analysis**

**Figure S7: Odds ratios of all loci that have an association p-value lower than 10e-5 in either cohort**

**Figure S8: p-values of association analysis in the iPSYCH2012 and iPSYCH2015 cohorts for all loci which are that have a p-value lower than 10e-5 in either cohort.** Each SNP is represented by a dot on the scatter plot, and each SNP has a p-value in the iPSYCH2015 cohort as well as a p-value in the iPSYCH2012 cohort. The SNPs in the plot are ones with p-value <10e-5 in either cohort.

**Figure S9: Tissue expression analysis of loci from the iPSYCH2012 genome-wide association analysis**

**Figure S10: Tissue expression analysis of loci from the iPSYCH2015 genome-wide association analysis**

**Figure S11: Manhattan plot of gene-based association analysis in the iPSYCH2012 cohort**

**Figure S12: Manhattan plot of gene-based association analysis in the iPSYCH2015 cohort.**

**Figure S13: GWAS analysis of phenotype stratified by disease severity in the iPSYCH2012 cohort**

**Figure S14: GWAS analysis of phenotype stratified by disease severity in the iPSYCH2015 cohort**

**Figure S15: GWAS meta-analysis of phenotype stratified by disease severity in the iPSYCH2012 and iPSYCH2015**

**Table S1.** **Gene set enrichment analysis of associated loci from the iPSYCH2012 GWAS analysis.** (See reference for further description of the gene sets in Molecular Signatures Database [30].)

| Gene Set | N genes | Beta | Beta STD | SE | p-value |
| --- | --- | --- | --- | --- | --- |
| Curated gene sets: stambolsky bound by mutated tp53 | 15 | 0.8245 | 0.025651 | 0.22929 | 0.00016227 |
| Curated gene sets: pid myc pathway | 21 | 0.68559 | 0.025232 | 0.19452 | 0.00021292 |
| GO bp: go regulation of phospholipase c activating g protein coupled receptor signaling pathway | 4 | 1.4107 | 0.022672 | 0.4115 | 0.00030471 |
| Go bp: go response to heparin | 5 | 1.4881 | 0.026737 | 0.43996 | 0.00036036 |
| Curated gene sets: chiaradonna neoplastic transformation cdc25 up | 97 | 0.31962 | 0.025219 | 0.094557 | 0.0003632 |
| Curated gene sets: enk uv response epidemics dn | 429 | 0.14662 | 0.024065 | 0.043563 | 0.00038284 |
| GO bp: go regulation of cysteine type endopeptidase activity involved in apoptotic signaling pathway | 16 | 0.70568 | 0.022673 | 0.21391 | 0.00048649 |
| Curated gene sets: Andersen cholangiocarcinoma class 2 | 140 | 0.24206 | 0.022913 | 0.073641 | 0.00050768 |
| Curated gene sets: reactome early phase hiv life cycle | 9 | 1.0564 | 0.025462 | 0.32452 | 0.00056784 |
| Curated gene sets: der ifn alpha response dn | 4 | 1.2743 | 0.020479 | 0.39917 | 0.0007075 |

**Table S2. Gene set enrichment analysis of associated loci from the GWAS analysis. (**See reference for further description of the gene sets in Molecular Signatures Database [30].)

| Gene Set | N genes | Beta | Beta STD | SE | P | Pbon |
| --- | --- | --- | --- | --- | --- | --- |
| GO bp: go head development | 632 | 0.15156 | 0.029765 | 0.036222 | 1.4388e-05 | 0.222539196 |
| GO mf: go supercoiled dna binding | 4 | 2.0336 | 0.032427 | 0.51932 | 4.5245e-05 | 0.69975917 |
| Curated gene sets: tsunoda cisplatin resistance dn | 36 | 0.57151 | 0.027311 | 0.1517 | 8.2859e-05 | 1 |
| GO bp: go forebrain development | 309 | 0.19257 | 0.026726 | 0.052238 | 0.00011416 | 1 |
| Curated gene sets: sakai chronic hepatitis vs liver cancer dn | 25 | 0.63479 | 0.025288 | 0.18317 | 0.00026537 | 1 |
| GO bp: go central nervous system development | 799 | 0.11187 | 0.24565 | 0.032526 | 0.0002925 | 1 |
| GO mf: go structural molecule activity conferring elasticity | 10 | 1.1406 | 0.028751 | 0.3354 | 0.00033701 | 1 |
| GO bp: go positive regulation of protein homoolihomerization | 9 | 1.0095 | 0.024141 | 0.29858 | 0.00036219 | 1 |
| GO mf: go extracellular matrix constituent conferring elasticity | 8 | 1.2643 | 0.028507 | 0.38032 | 0.00044442 | 1 |
| GO bp: go negative regulation of signaling | 1089 | 0.092094 | 0.023379 | 0.027774 | 0.00045804 | 1 |

**Table S3. Top ten associated SNPs among severe cases in the iPSYCH2012 cohort**

| SNPs | Chromosome | Bp | P | OR | Gene |
| --- | --- | --- | --- | --- | --- |
| rs34341914 | 5 | 114500839 | 9.60493e-07 | 0.0308049 | TRIM36 |
| rs79069767 | 5 | 114510834 | 5.14269e-07 | 0.0317470 | TRIM36 |
| rs6963269 | 7 | 19380375 | 5.76035e-07 | 0.0150829 |  |
| rs10156000 | 7 | 19381241 | 1.52630e-06 | 0.0145058 |  |
| rs2159742 | 7 | 19382024 | 1.51430e-06 | 0.0145104 |  |
| rs2109847 | 7 | 19382301 | 1.47894e-06 | 0.0145262 |  |
| rs10265518 | 7 | 19384131 | 1.41460e-06 | 0.0148678 |  |
| rs6945700 | 7 | 19389603 | 1.53179e-06 | 0.0147681 |  |
| rs6945594 | 7 | 19389750 | 1.20227e-06 | 0.0149280 |  |
| rs4803930 | 19 | 46827829 | 3.89243e-07 | -0.0196404 | HIF3A |

**Table S4. Top ten associated SNPs among severe cases in the iPSYCH2015 cohort**

| SNPs | Chromosome | Bp | P | OR | Gene |
| --- | --- | --- | --- | --- | --- |
| rs4300273 | 1 | 95961209 | 5.23793e-06 | 0.0263353 |  |
| rs12071306 | 1 | 95968479 | 3.98335e-06 | 0.0266084 |  |
| rs4949966 | 1 | 95969035 | 4.05381e-06 | 0.0265857 |  |
| rs829002 | 1 | 108700316 | 4.77812e-06 | 0.0209909 | SLC25A24 |
| rs1069909 | 1 | 108708570 | 5.30038e-06 | 0.0208834 | SLC25A24 |
| rs829008 | 1 | 108713005 | 5.30038e-06 | 0.0208834 | SLC25A24 |
| rs557718 | 1 | 108716399 | 5.30038e-06 | 0.0208834 | SLC25A24 |
| rs597186 | 1 | 108726246 | 5.3346e-06 | 0.0208797 | SLC25A24 |
| rs34297043 | 1 | 160677613 | 2.14821e-06 | 0.0298309 | CD48 |
| rs696824 | 9 | 79262737 | 2.63522e-06 | -0.0234546 | PRUNE2 / LOC105376095 |
