# Supplementary figures and images for: Genome-wide Association Study of Susceptibility to Respiratory Syncytial Virus Hospitalization in Young Children <5 Years of age
Source: J Infect Dis. 2023 Sep 4;230(2):e333–41. doi: 10.1093/infdis/jiad370 (PMC11326809; doi:10.1093/infdis/jiad370)

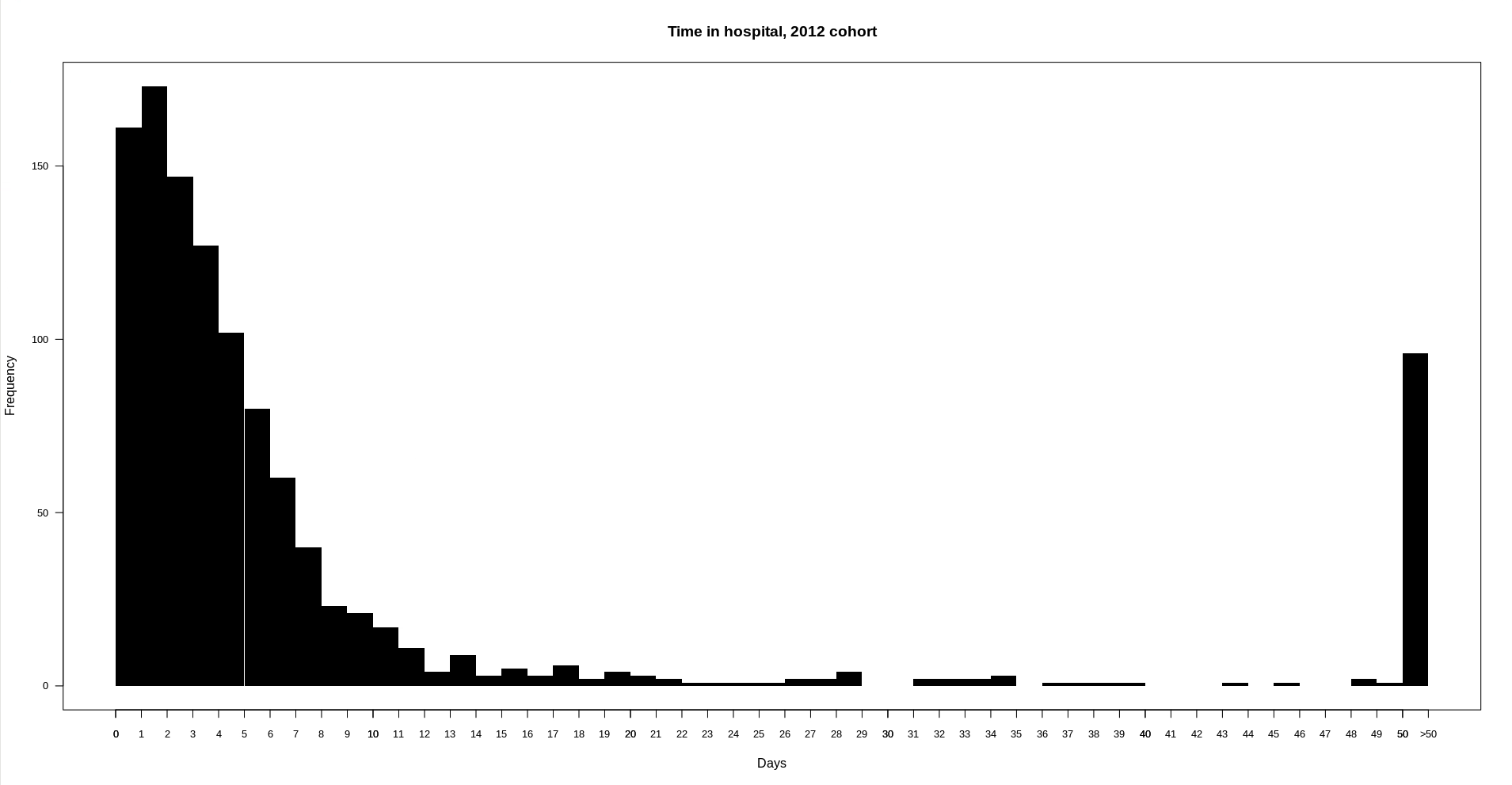

Supplement: jiad370_Supplementary_Data [file jiad370_supplementary_data.zip › Figure S1.tif]

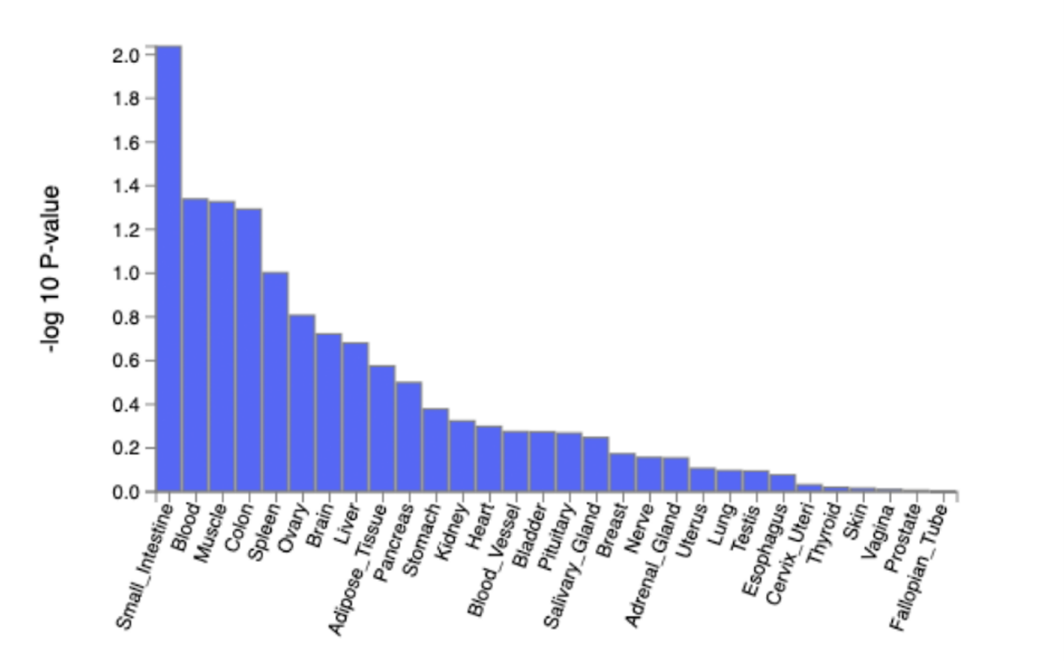

Supplement: jiad370_Supplementary_Data [file jiad370_supplementary_data.zip › Figure S10.tif]

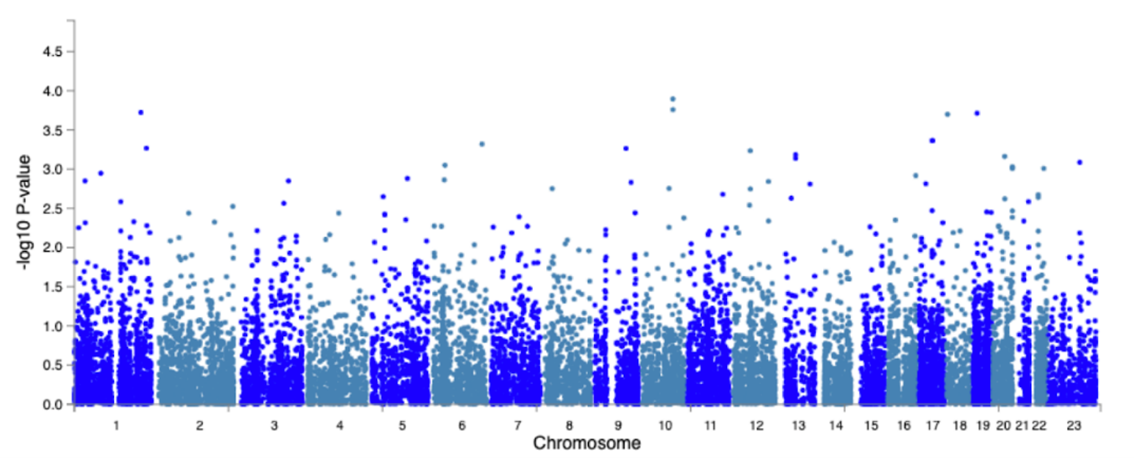

Supplement: jiad370_Supplementary_Data [file jiad370_supplementary_data.zip › Figure S11.tif]

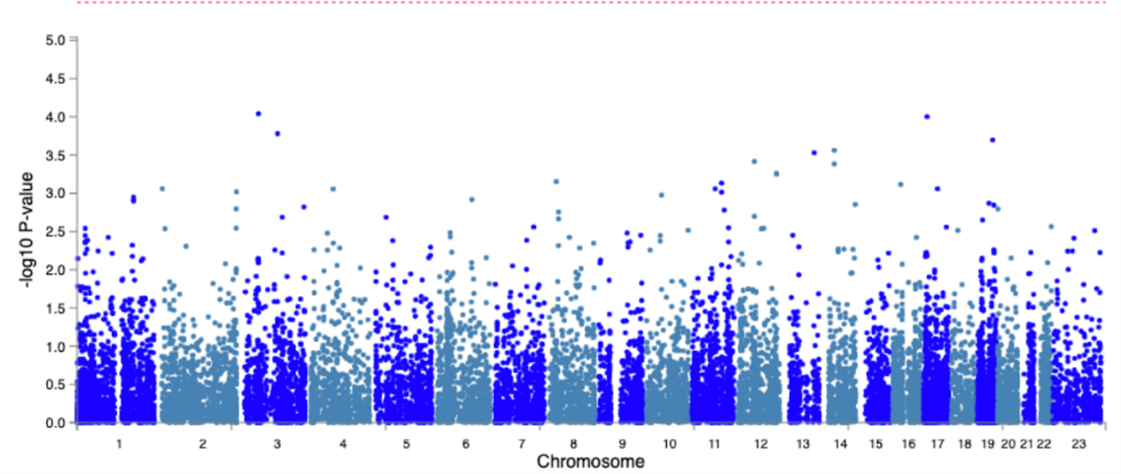

Supplement: jiad370_Supplementary_Data [file jiad370_supplementary_data.zip › Figure S12.tif]

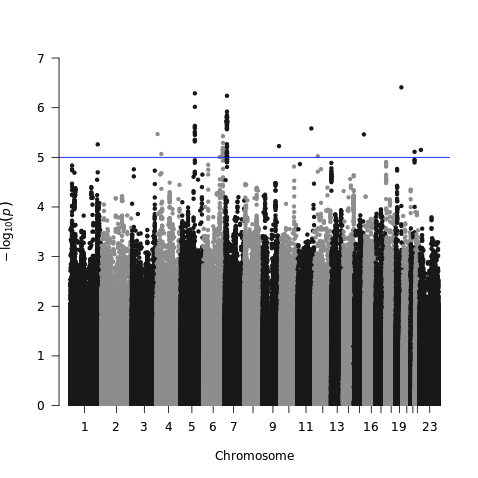

Supplement: jiad370_Supplementary_Data [file jiad370_supplementary_data.zip › Figure S13.tif]

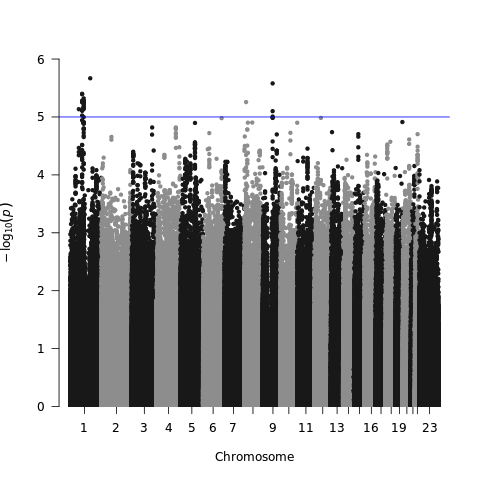

Supplement: jiad370_Supplementary_Data [file jiad370_supplementary_data.zip › Figure S14.tif]

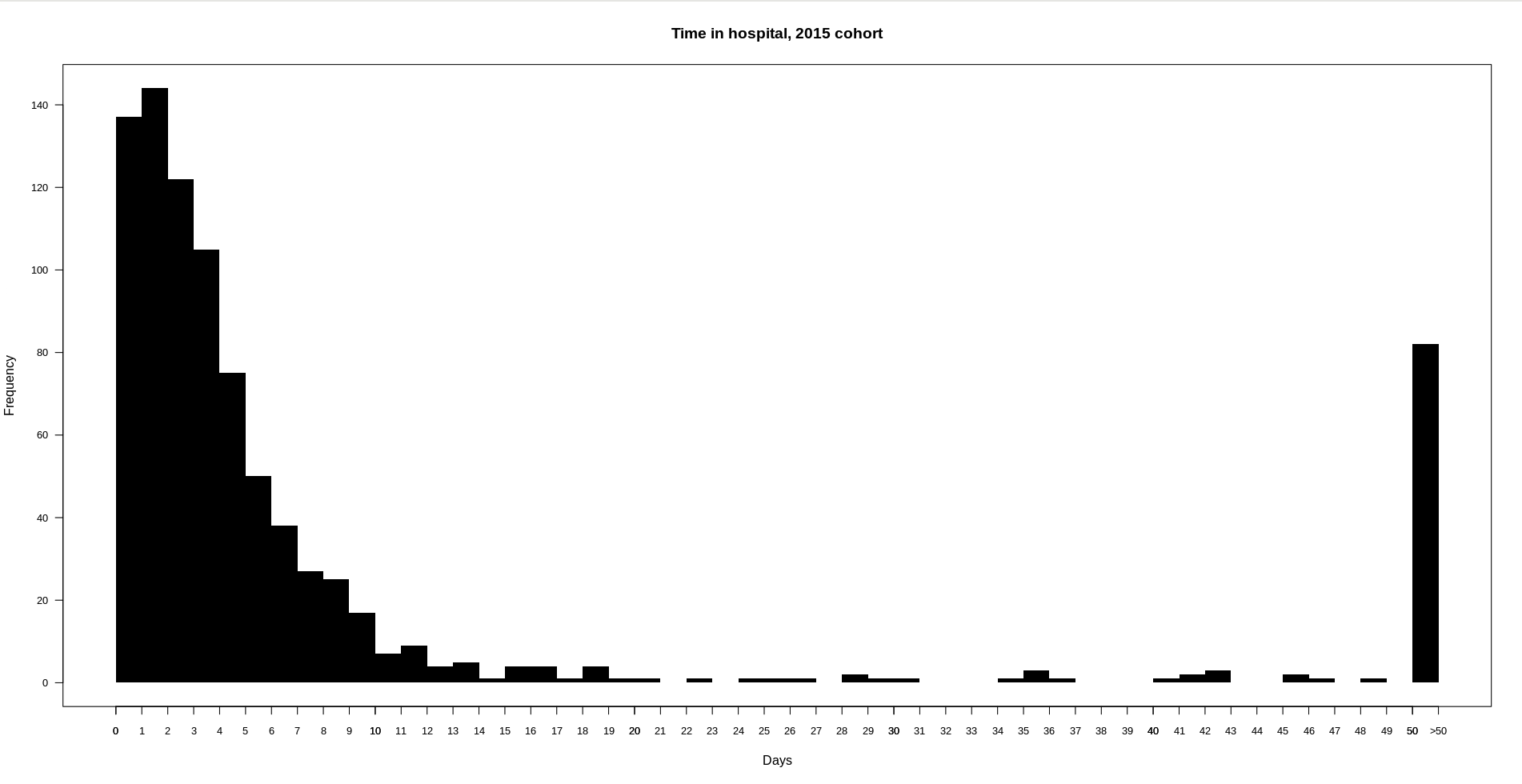

Supplement: jiad370_Supplementary_Data [file jiad370_supplementary_data.zip › Figure S2.tif]

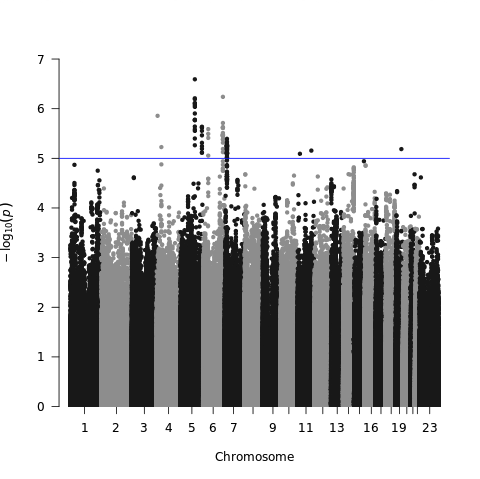

Supplement: jiad370_Supplementary_Data [file jiad370_supplementary_data.zip › Figure S3.tif]

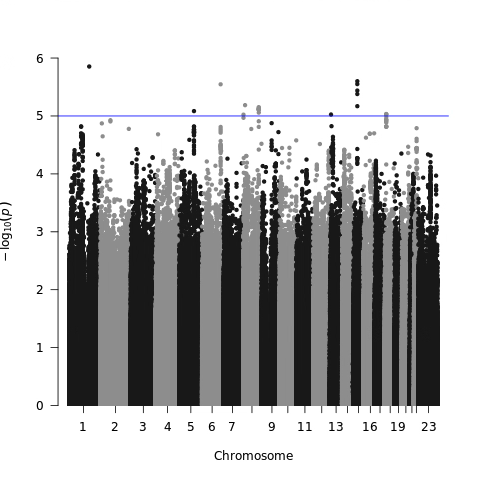

Supplement: jiad370_Supplementary_Data [file jiad370_supplementary_data.zip › Figure S4.tif]

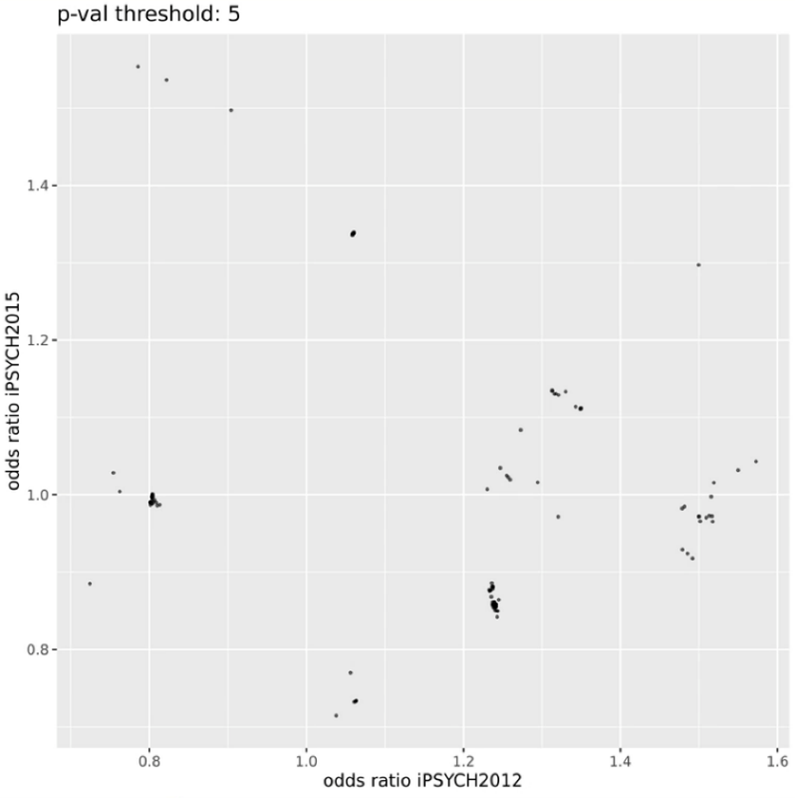

Supplement: jiad370_Supplementary_Data [file jiad370_supplementary_data.zip › Figure S7.tif]

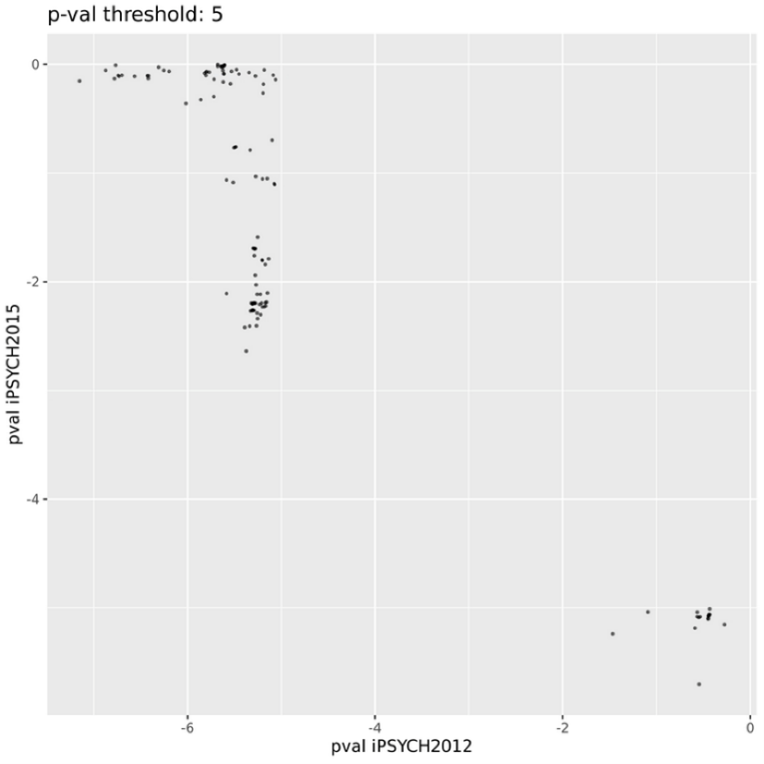

Supplement: jiad370_Supplementary_Data [file jiad370_supplementary_data.zip › Figure S8.tif]

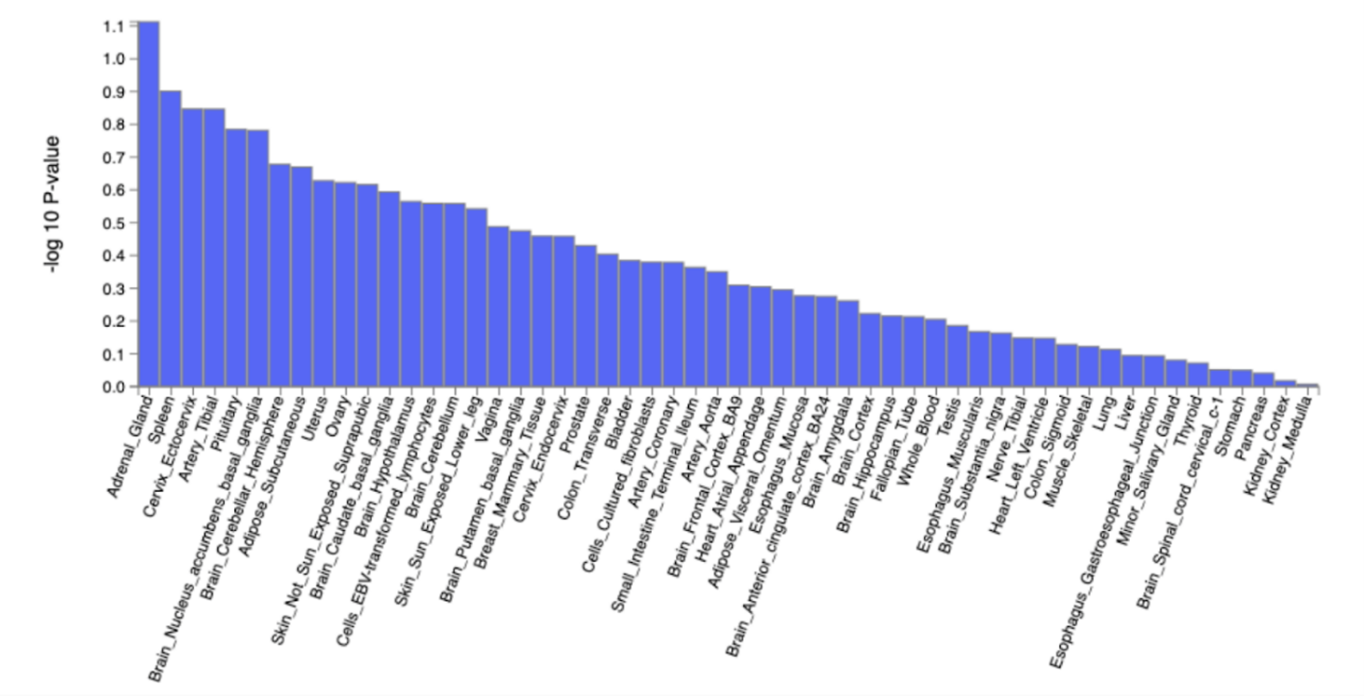

Supplement: jiad370_Supplementary_Data [file jiad370_supplementary_data.zip › Figure S9.tif]

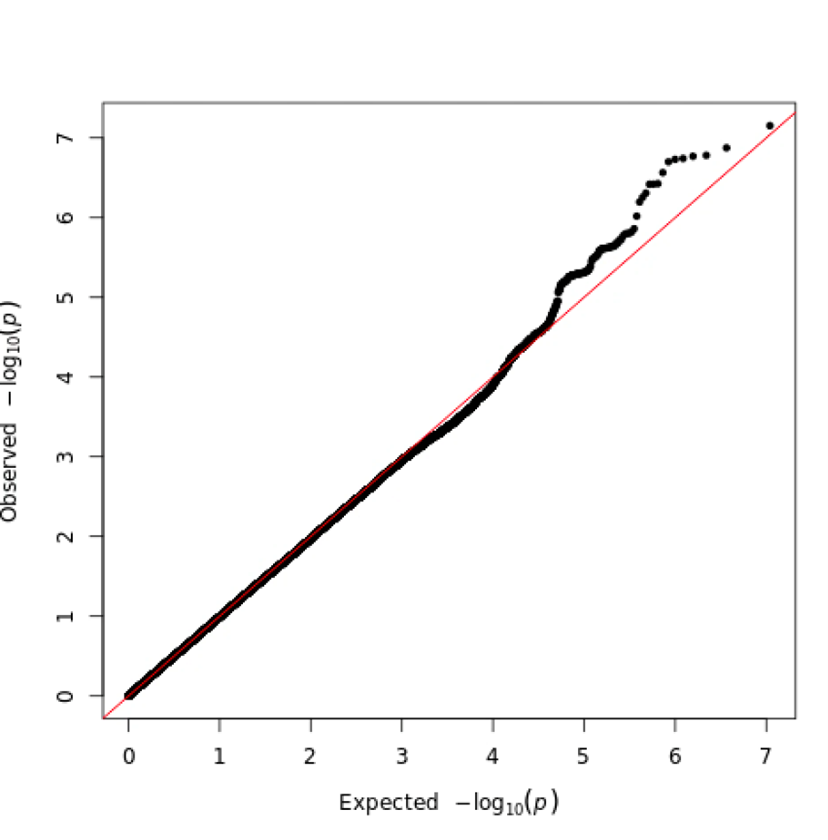

Supplement: jiad370_Supplementary_Data [file jiad370_supplementary_data.zip › FigureS5.tif]

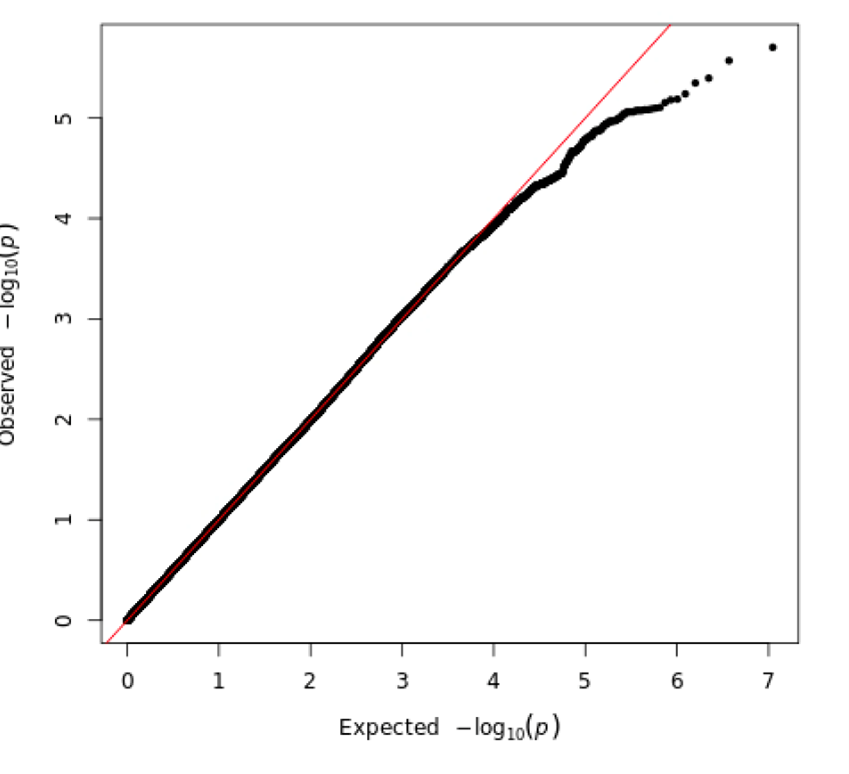

Supplement: jiad370_Supplementary_Data [file jiad370_supplementary_data.zip › FigureS6.tif]
